# Geometry-Based versus Small-Molecule Tracking Method for Tunnel Identification: Benefits and Pitfalls

**DOI:** 10.1021/acs.jcim.2c00985

**Published:** 2022-11-14

**Authors:** Karolina Mitusińska, Maria Bzówka, Tomasz Magdziarz, Artur Góra

**Affiliations:** Tunneling Group, Biotechnology Centre, Silesian University of Technology, Krzywoustego 8, 44-100 Gliwice, Poland

## Abstract

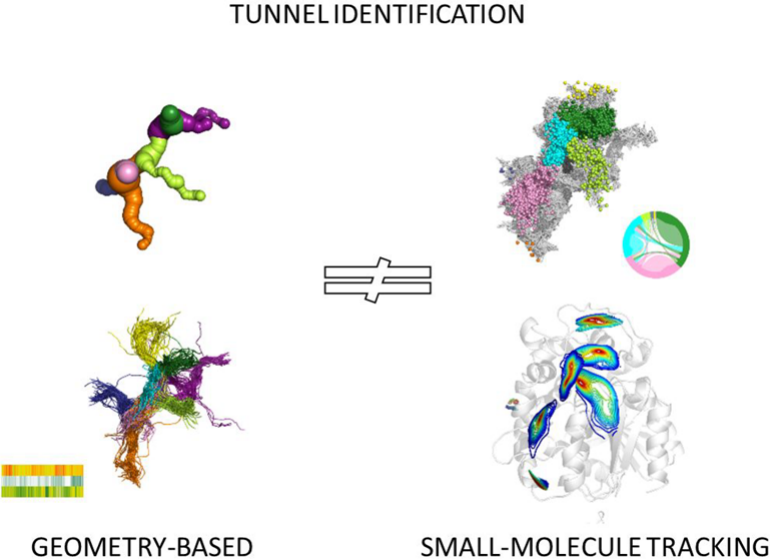

Different methods for tunnel identification, geometry-based and small-molecule tracking approaches, were compared to provide their benefits and pitfalls. Results obtained for both crystal structures and molecular dynamics (MD) simulations were analyzed to investigate if a more computationally demanding method would be beneficial. Careful examination of the results is essential for the low-diameter tunnel description, and assessment of the tunnel functionality based only on their geometrical parameters is challenging. We showed that the small-molecule tracking approach can provide a detailed description of the system; however, it can also be the most computationally demanding.

## Introduction

Most bioinformatics workflows start with an application of a simple approach providing a general description of the problem followed by the application of more complex and time-consuming solutions that guarantee a deeper understanding of the described phenomena. The same pipeline is observed in structural biology studies, such as tunnel identification in protein structure.^1^ In our study, a tunnel is defined as a pathway connecting the protein surface with an internal cavity or a pathway connecting more than one cavity (definition taken from Prokop et al.^2^). Tunnels gain significant importance due to the set of functions they maintain in enzymes, i.a., control of the activity and selectivity and reaction synchronization.^3−5^ More than half of currently known protein structures are equipped in tunnels; therefore, tunnel identification is carried out as a standard procedure, especially in enzymes with buried active sites.^6^

First and still the most commonly used approach for tunnel identification is the geometry-based approach reviewed in ref (1). This approach employs the construction of a Voronoi diagram to detect and describe voids within a macromolecule structure. Then, tunnels are identified using a predefined probe radius and internal “empty spaces”. However, this approach is usually used to analyze crystal structures or single molecular dynamics (MD) simulation snapshots. This approach was implemented in different software, such as CAVER 3.0, MOLE 2.0, MolAxis, or ChExVis (which do not differ substantially as shown by Brezovsky et al.^1^). Of those geometry-based methods, only CAVER 3.02 analyzes the whole MD simulation, which provides a general overview of the potential tunnels connecting the active site with the enzyme’s environment. CAVER 3.02 is applied to a series of frames derived from MD simulations where the dynamic of tunnel-lining residues is taken into account. In each analyzed snapshot (the user can select which frames they want to analyze further), tunnels are identified based on the diameter of the defined probe. The clustering algorithm implemented in CAVER 3.02 is applied to compare and group identified tunnels, and thus, the tunnel opening and closing events can be observed.^7^ Still, this approach considers only the geometry of the tunnels, while the physical and chemical properties of potential molecules transported via tunnels are neglected. This simplification may not be considered an obstacle if there is only one tunnel leading to the active center. However, this is not always the case.^4,8^ The choice of the transport pathway for a given substrate/product is no longer trivial in the case of multiple tunnels connecting the active center to the environment. Tunnels in proteins maintain different functions, such as transport of ligand, product, solvent, and/or ions to and from the active site. Description of a tunnel’s functionality, even *in silico*, is a complex process that requires lots of computational efforts, such as MD simulations combined with tracking of small molecules through the tunnels.^4,8,9^

A different approach to tunnel identification has been proposed by the developers of the AQUA-DUCT software.^10,11^ The software uses a small-molecule tracking approach to provide information on the flow direction and tunnel contribution during MD simulations. AQUA-DUCT traces water molecules (or other selected small molecules present in the simulated system) penetrating the protein’s interior. Thus, in contrast to the geometry-based methods, it includes physicochemical properties of the tunnel-lining residues and identifies only those tunnels which are capable of transporting water or other small molecules of interest. However, this approach requires analysis of bigger files (MD simulation trajectory files consisting of protein and solvent molecules) and relatively large sampling (in terms of the number of frames) of MD simulations to draw and analyze the pathways of the analyzed small molecules (for benchmark of the AQUA-DUCT resource usage and effect of the trajectory time-step on the obtained results, see ref (11)). Therefore, the small-molecule tracking approach may be more demanding compared with the geometry-based approach in terms of preparing the MD simulation trajectory files and their storage.

So far, no comparison of the above-mentioned approaches has been made. An extensive comparison of the geometry-based methods was made by Brezovsky et al.,^1^ in which they also stressed the existing limitations of those types of analysis, such as lack of information on electrostatics, hydrophobicity, or dynamics of identified pathways. However, it remains unknown whether it is beneficial to use outcomes of the MD simulations or if the analysis of crystallographic structures is sufficient. In this study, we collated the profits of using more advanced tools with the oversights or misinterpretations of using the simplest techniques. As a model system, we chose representative members of the soluble epoxide hydrolases (sEH), a group of enzymes which belong to the α/β-hydrolases fold family,^12−14^ due to their diverse tunnel network.^15^ We hope that our results shed light on tunnel identification in protein structure and the interpretation of the results and will help researchers with an adequate selection of the method corresponding with their requirements and expectations.

## Materials and Methods

### Obtaining Protein Structures for Analysis

Eight unique and complete crystal structures were downloaded from the Protein Data Bank (PDB)^16^ representing the same set of structures as used elsewhere:^15,17^*Homo sapiens* (hsEH, PDB ID: 1S8O), *Mus musculus* (msEH, PDB ID: 1CQZ), *Solanum tuberosum* (StEH1, PDB ID: 2CJP), *Vigna radiata* (VrEH2, PDB ID: 5XM6), (*Trichoderma reesei* (TrEH, PDB ID: 5URO), *Bacillus megaterium* (bmEH, PDB ID: 4NZZ), and two structures from an unknown source organism collected from hot springs in Russia and China (Sibe-EH, PDB ID: 5NG7; CH65-EH, PDB ID: 5NFQ).

### MD Simulations

The H++ server^18^ was used to protonate the analyzed structures using standard parameters at the reported optimal pH for the enzyme activity (Table S1). Counterions were added to the structures to neutralize the systems. Water molecules were placed using the combination of 3D-RISM theory^19^ and Placevent algorithm.^20^ The Amber14 tLEaP^21^ package was used to immerse the models in a truncated octahedral box with a 10 Å radius of TIP3P water molecules, and the ff14SB force field^22^ was used for parametrization of each system. A PMEMD CUDA package of Amber14 software was used to run a single repetition of a 50 ns MD simulation of selected sEHs. The minimization procedure consisted of 2000 steps, involving 1000 steepest descent steps followed by 1000 steps of conjugate gradient energy minimization with decreasing constraints on the protein backbone (500, 125, and 25 kcal × mol^–1^ × Å^–2^) and a final minimization with no constraints of conjugate gradient energy minimization. Next, gradual heating was performed from 0 to 300 K over 20 ps using a Langevin thermostat with a collision frequency of 1.0 ps^–1^ in periodic boundary conditions with constant volume. The equilibration stage was conducted using the periodic boundary conditions with constant pressure for the time stated in Table S1 with a 1 fs time step using Langevin dynamics with a collision frequency of 1.0 ps^–1^ to maintain a constant temperature. The production stage was conducted for 50 ns with a 2 fs time step using Langevin dynamics with a collision frequency of 1.0 ps^–1^ to maintain a constant temperature. Long-range electrostatic interactions were modeled using the particle mesh Ewald method with a nonbonded cutoff of 10 Å and SHAKE algorithm. The coordinates were saved at 1 ps intervals. The number of added water molecules and ions is shown in Table S1.

### Tunnel Identification: CAVER Analysis

Tunnel identification and analysis in each system was carried out using CAVER software^23^ in two steps: (i) the crystal structure of the enzyme was analyzed by the CAVER plugin for PyMOL;^23^ (ii) tunnels were identified and analyzed in 50,000 snapshots of multiple MD simulations by CAVER 3.02 software.^23^ Parameters used for both steps are shown in Table S2. The tunnels found during MD simulations and in crystal structures were ranked and numbered on the basis of their throughput value.^23^

### Tunnel Identification: AQUA-DUCT Analysis

AQUA-DUCT analysis was carried out according to the protocol described elsewhere.^15,24^ A small-molecule tracking approach implemented in AQUA-DUCT^10,11^ was used for tunnel identification and assessment of their functionality. Tunnel’s functionality was defined as the ability of the tunnel to transport small molecules (such as water molecules, ions, ligands, or cosolvents, such as methanol, phenol, etc.).

### Tunnels Comparison

Tunnels were identified in both crystal structures and during MD simulations and then compared with each other to find their corresponding counterparts. First, the tunnels identified during MD simulations and in crystal structures were maintained using the same approach as described elsewhere.^17^ In the case of tunnels identified in MD simulations by CAVER 3.02 but for which no corresponding counterpart was found in the crystal structures by CAVER plugin for PyMOL, their tunnel-lining residues were selected based on the cutoff threshold of 0.65. This value was chosen on the basis of quantile computations for the tunnels identified in MD simulation, which had their counterparts in the crystallographic structures.

Tunnel functionality was then assessed based on a small-molecule tracking approach implemented in AQUA-DUCT by superposing the paths of water molecules, and their entry/exit areas with tunnels were identified by CAVER in both the crystal structures and during MD simulations. A visual comparison allowed matching of the water molecule pathways and tunnels identified by CAVER.

## Results

**Figure 1:**
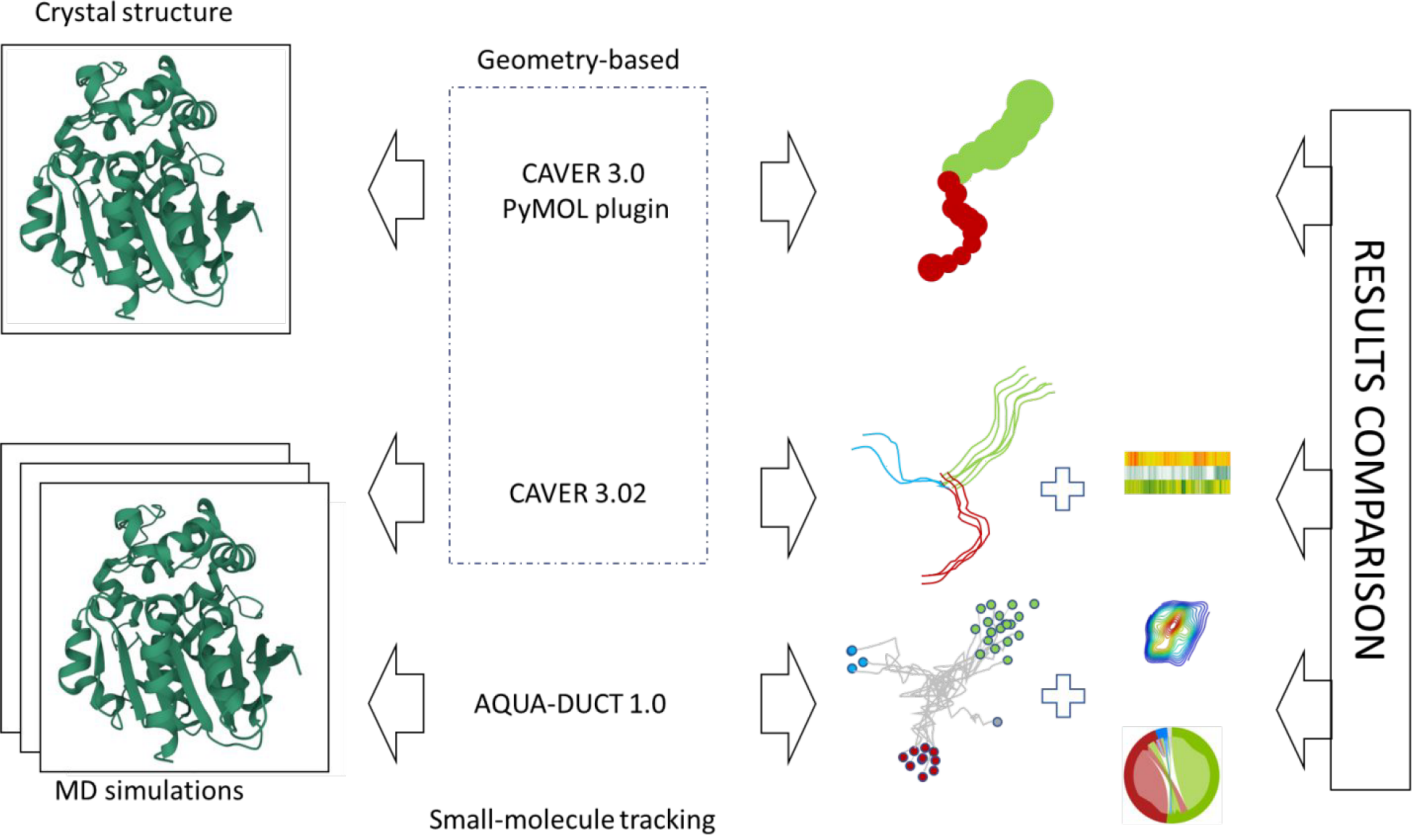
Tunnel identification and schematic comparison of the obtained results: (upper row) CAVER 3.0 PyMOL plugin, tunnel visualization in crystal structures; (middle row) CAVER 3.02, centerlines of the identified tunnels and tunnel occurrence heatmap; (bottom row) AQUA-DUCT, inlet clusters with water molecule pathways, entry/exit areas, and intramolecular flowchart.

Here, we chose the same set of eight sEHs as presented in ref (15). We mimicked a typical approach used in various studies regarding tunnel identification in protein structure (Figure 1). The terms tunnel and channel are often used interchangeably in the scientific literature; therefore, based on Prokop et al.,^2^ we used unifying terminology: that a tunnel is a pathway connecting the protein surface with an internal cavity or a pathway connecting two or more cavities. A channel is then a pathway leading throughout the protein structure without any interruption by an internal cavity with both sides open to the surrounding solvent. We started with a simple analysis of crystal structures downloaded from Protein Data Bank using a geometry-based tool, CAVER 3.0 PyMOL plugin, which is one of the most widely used tools for tunnel identification. Then, we expanded our analysis to tunnel identification during MD simulations. We used CAVER 3.02 software to analyze a single repetition of MD per protein, as is often the case in other studies. CAVER 3.02 is based on the same principles as its plugin counterpart with the advantage of taking into account the information from MD simulations. Lastly, we used AQUA-DUCT 1.0, which uses the small-molecule tracking approach during MD simulations. As an input, we used the same MD simulations as were used during CAVER 3.02 analysis. Thus, for each structure, we obtained results from three different approaches: (i) geometry-based approach applied on a crystal structure, (ii) geometry-based approach applied on an MD simulation, and (iii) small-molecule tracking approach applied on an MD simulation. A comparison of these results (Tables 1 and S2–S9) provided insights on when it is best to use a particular approach as well as their benefits and pitfalls and how they can bias the bigger picture.

**Table 1:** Comparison of Results Obtained by the Geometry-Based and Small-Molecule Tracking Approaches in the Crystal Structures and during MD Simulations^a^

		CAVER 3.0 PyMOL plugin		CAVER 3.02			AQUA-DUCT	
		crystal structure		MD simulations				
enzyme	tunnel	rank	max_bottleneck [Å]	rank	max_bottleneck [Å]	occurrence [%]	inlets	cluster area [Å^2^]
hsEH	Tc/m1	1	1.58	2	2.70	59	554 (22%)	67.0
	Tm1	2	1.78	1	2.87	100	1830 (79%)	93.2
	Tg	3	1.10	8	1.86	11	94 (4%)	82.5
	Tc/m2	4	1.34	15	1.96	1		
	Tm5	5	1.18	5	1.82	54	1 (<1%)	
	Tcap4	6	1.15	16	1.34	1		
	Tcap2b	7	0.95	7	1.52	19	3 (<1%)	1.4
	Tm3	8	0.92	14	1.11	2		
	Tm2			3	2.57	95	24 (1%)	15.7
	Tc/m_side			14	2.00	48	1 (<1%)	
	Tcap1						7 (<1%)	46.5
msEH	Tc/m1	1	2.09	1	3.18	99	1627 (38%)	70.7
	Tm1	2	2.01	2	3.14	100	1400 (32%)	77.7
	Tcap4	3	1.12	6	1.93	51	6 (<1%)	11.7
	Tm2	4	1.03	5	2.23	57	7 (<1%)	21.7
	Tm3	5	1.02	3	2.37	88	215 (5%)	108.9
	Tside	6	0.96	9	1.21	11	5 (<1%)	
	Tg			4	2.96	71	1031 (24%)	46.5
	Tcap1			7	2.61	15	33 (1%)	86.4
TrEH	Tc/m1	1	2.40	1	2.93	96	986 (41%)	234.7
	Tm1	2	1.45	2	2.65	100	1200 (54%)	212.3
	Tcap4	3	1.28	3	1.95	53	23 (1%)	41.9
	Tside	4	0.96	4	2.33	53	10 (<1%)	26.5
	Tm5	5	0.91	6	1.81	12		
	Tback	7	0.91	13	1.1	2		
	Tc/m_back						1 (<1%)	
StEH1	Tm1	1	1.79	1	3.07	100	1774 (89%)	149.4
	Tc/m1	2	1.40	3	2.11	98	149 (7%)	7.8
	Tm2	3	1.79	4	2.52	65	65 (3%)	112.0
	Tcap3	4	1.14	12	1.42	13	1 (<1%)	
	Tcap6	5	0.97	7	1.36	28		
	Tc/m_back	6	1.10	16	1.28	8		
	Tcap5	7	0.93	9	1.44	19		
	Tm5			6	2.04	21	6 (<1%)	11.8
VrEH2	Tm1	1	1.41	1	2.84	89	563 (93%)	68.5
	Tcap1	2	1.30	4	1.61	53	1 (<1%)	
	Tside	3	1.31	5	1.51	53		
	Tm5	4	1.14	2	1.98	80	10 (2%)	24.8
	Tm2			3	2.00	77	27 (4%)	30.9
	Tcap2b			7	1.51	30	1 (<1%)	
	Tcap7			13	1.49	7	1 (<1%)	
bmEH	Tc/m1	2	1.92	1	2.74	100	5256 (100%)	88.4
	Tc/m_back	3	1.06	3	1.88	18		
	Tcap7	4	0.90	4	1.17	1		
CH65-EH	Tc/m1	1	1.45	2	2.29	97	3375 (88%)	67.29
	Tc/m_back	2	1.56	1	2.48	100		
	Tc/m_side	3	1.19	5	1.54	28		
	Tm4			4	2.06	46	359 (9%)	126.1
	Tcap4			6	1.72	25	84 (2%)	104.9
Sibe-EH	Tc/m1	1	1.89	1	2.51	100	1011 (98%)	30.1
	Tc/m3	2	1.11	3	1.88	23		
	Tc/m_back	3	1.16	9	1.20	4	20 (2%)	86.5
	Tcap4	4	1.05	6	1.70	14	1 (<1%)	
	Tc/m2	5	1.91	12	1.44	2		
	Tside	6	0.91	13	1.24	2	1 (<1%)	

a Please note that the table comprises the best matches between tunnels identified in crystal structures and during MD simulations. The detailed tunnel comparison results are provided in Tables S3–S10.

### Tunnels Identification in Crystal Structures by CAVER 3.0 PyMOL Plugin

**Figure 2:**
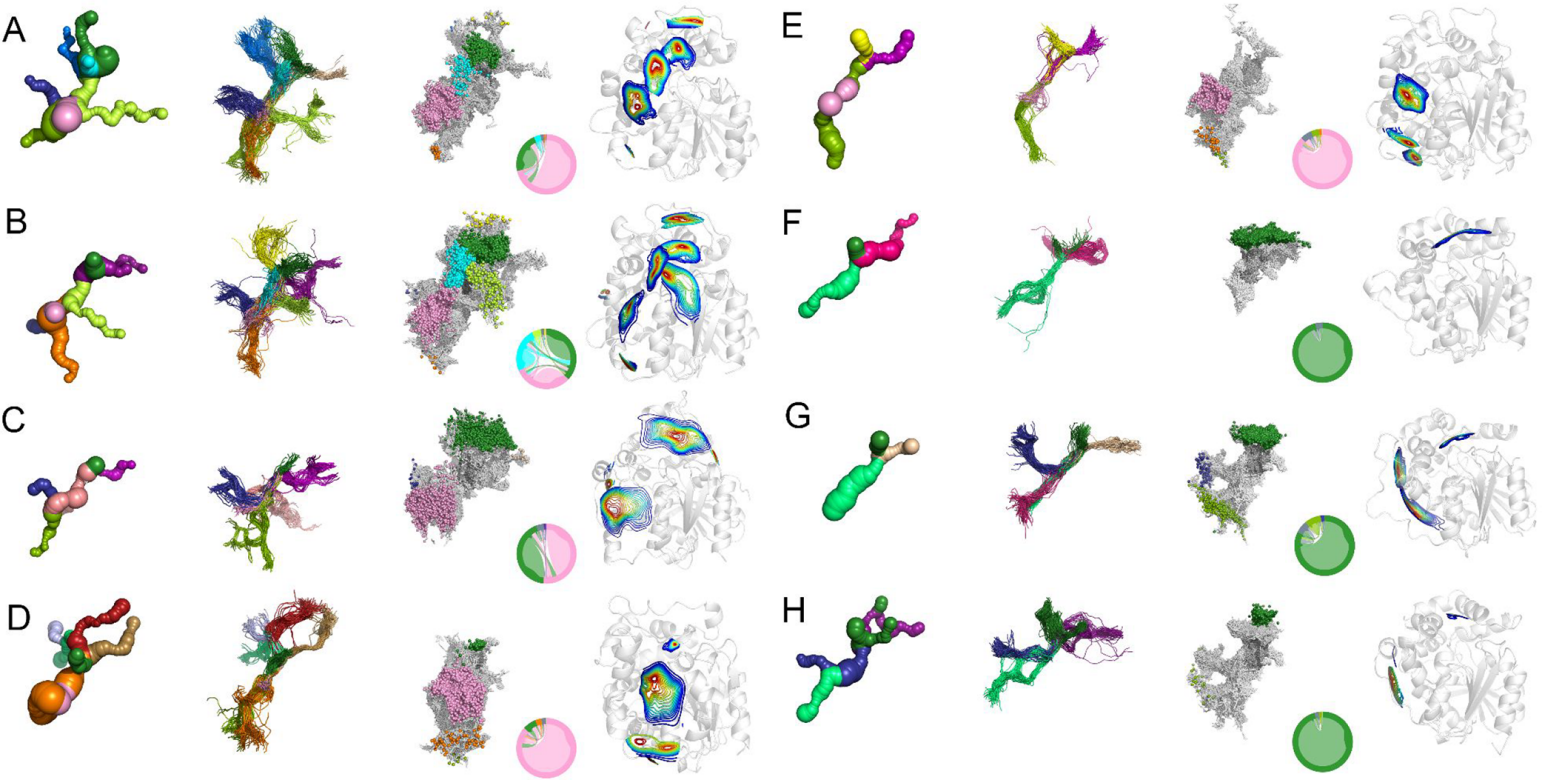
Comparison of results obtained from the geometry-based and small-molecule tracking approaches for the following epoxide hydrolases: (A) *Homo sapiens* (hsEH), (B) *Mus musculus* (msEH), (C) *Trichoderma reesei* (TrEH), (D) *Solanum tuberosum* (StEH1), (E) *Vigna radiata* (VrEH2), (F) *Bacillus megaterium* (bmEH), and (G) Sibe-EH and (H) CH65-EH identified in hot springs. Each panel comprises tunnels obtained by the CAVER 3.0 PyMOL plugin for the crystal structure, tunnel centerlines obtained by CAVER 3.02 software for molecular dynamics (MD) simulations, and inlet clusters with water molecule pathways, entry/exit areas, and an intramolecular flowchart obtained by AQUA-DUCT from MD simulations. Corresponding tunnels, centerlines, and inlet clusters are marked with the same color; entry/exit areas are colored according to their density: blue represents the overall shape of the entry/exit area and red, the region in which the highest number of inlets was identified.

The simplest approach aims to identify tunnels in crystal structures. The CAVER 3.0 PyMOL plugin provides information about the number of tunnels, their length, and bottleneck radius. In our study, it identified three (in CH65-EH) to nine (in hsEH) tunnels in the analyzed protein structures with the maximal bottleneck ranging from 0.9 Å in bmEH to 2.4 Å in the TrEH structure (Figure 2, Table 1). We used the same naming for the identified tunnels as in our previous studies,^15,17^ based on the region in which the tunnel was identified (Tcap, for tunnels found in the cap domain; Tm, for tunnels identified in the main domain; Tc/m, for tunnel identified at the border between both domains). The detailed list of tunnels identified in the crystal structures is in Table 1.

### Tunnels Identification in MD Simulations by CAVER 3.02

We ran a single repetition of MD simulations for each sEH and analyzed them using CAVER 3.02 software, which processed a set of snapshots from an MD simulation and identified tunnels in each of them. Then, CAVER 3.02 performed clustering on tunnels which it considers similar; i.e., tunnels whose portions lead through the same part of the structure. Clustering provided a clear picture of tunnels in the same conformation. The number of tunnels identified by the CAVER 3.02 software in an MD simulation was often higher than the number of tunnels identified by the CAVER 3.0 PyMOL plugin in the crystal structure (Figure 2, Table 1). This is due to the formational changes which proteins undergo. Comparison of tunnels and selection of the best corresponding counterparts (Tables S3–S10) were done based on the similarity of tunnel-lining residues (see Materials and Methods section for a detailed description of the comparison procedure).

Additionally, CAVER 3.02 provided information on the tunnel’s occurrence, which is measured as the number of frames in which the tunnel was identified. In most structures, except VrEH2, at least one tunnel was identified as open during the whole simulation time. In four structures, msEH, hsEH, TrEH, and StEH1, the Tm1 tunnel was identified as the always open tunnel, while for bmEH and Sibe-EH, it was the Tc/m tunnel and for CH65-EH, the Tc/m_back tunnel. In the case of VrEH2, the most often open tunnel was the Tm1 tunnel; it was open for 89% of the simulation time. However, it is worth noting that in the case of msEH, StEH1, CH65-EH, TrEH, and hsEH the second most often tunnel is the Tc/m tunnel, which is open for 99%, 98%, 97%, 96%, and 59% of the simulation time, respectively (Table 1).

### Tunnel Entrance/Exit Area Identification in MD Simulations by AQUA-DUCT

The same MD simulations were examined using AQUA-DUCT to identify the tunnel entrance/exit areas using the small-molecule tracking approach. AQUA-DUCT traced water molecules which entered and/or left the active site cavities of sEHs. Thus, it identified tunnels which were actually used by water molecules, which we will be referring to as the functional tunnels.

AQUA-DUCT identified one (in bmEH) to nine (in msEH) functional tunnels in the analyzed sEHs (Figure 2, Table 1). Tunnels were named using the previously established scheme regarding their exit location in the sEH structure. It should be noted that some tunnels found by CAVER do not have their functional counterparts identified by AQUA-DUCT. The opposite situation, when tunnels identified by the small-molecule tracking approach do not have their counterparts identified by CAVER, also occurred.

While CAVER 3.02 software provided the tunnel’s occurrence, AQUA-DUCT provided the information on the number of inlets identified in each entrance/exit area. An inlet is a representation of the point in which a traced molecule entered or left the active site cavity. It can be assumed that the main tunnel will transport the highest number of water molecules, i.e., will have the highest number of inlets. The distribution of inlets approximated the shape and size of the tunnel entry/exit area (Figure 2). Moreover, the intramolecular flow plot provided information regarding the water molecules’ exchange and flow direction (Figure 2). According to AQUA-DUCT results, the sEHs can be divided into three groups: (i) in which the Tc/m1 tunnel was the main tunnel (bmEH, CH65-EH, and Sibe-EH), (ii) in which the Tm1 was the main tunnel (StEH1 and VrEH2), and (iii) in which the Tc/m1 and Tm1 were the main tunnels (msEH, hsEH, and TrEH).^15^

## Discussion

Computational identification of tunnels in proteins, based on their crystal structures, has been performed since the beginning of the 21st century.^1^ With the introduction of MD simulations, this capability was soon extended.^23,25^ However, the vast majority of previously investigated tunnels have been described on the basis of solely crystal structures. While a crystal structure provides information on a single potential protein conformation, MD simulations provide a more detailed picture of protein motion and conformational changes. However, it should be kept in mind that the obtained picture depends on the force field and/or water models used during the MD simulation, which is out of the scope of our research. Importantly, the assessment of tunnel functionality still remains a nontrivial task due to several reasons, e.g., (i) variety of functions maintained by different tunnels in a protein (substrate entry, product egress, solvent accessibility control) and (ii) lack of a direct experimental method for small molecule transport assessment. Only indirect analyses can be provided, which require mutant design and kinetic studies supported by advanced in silico methods as shown by Biedermannová et al.^26^ The mentioned study suggests that results derived by advanced computational methods such as combination of Random Acceleration Molecular Dynamics (RAMD) and Adaptive Biasing Force (ABF) can be approximated by the geometry-based methods. So far, the performance of the recently developed small-molecule tracking method was not compared with commonly used approaches. We would like to point out that our research presents the first ever reported comparison of the geometry-based and small-molecule tracking approaches for tunnel identification in proteins. We hope that our results will help researchers to adequately select the method corresponding to their requirements and expectations. A comparison of the results obtained using both approaches on sEHs provided a systematic overview of the benefits and pitfalls of those methods.

### Comparison of Results Obtained with Geometry-Based Approach in Crystal Structures and MD Simulations

Our results suggest that most tunnels identified during MD simulations have their counterparts in tunnels identified in crystal structures. However, closer inspection of the systems chosen for our study shows that reported tunnel shape and size can differ substantially in some cases (Figure S1). Differences may be attributable to packing inaccuracies or poor resolutions of crystal structures.^27−30^ Here, the structure with the poorest resolution (msEH, 2.80 Å resolution) also had the highest average difference measured between bottlenecks in corresponding tunnels identified in the crystal structure and during the MD simulation. Crystal structures with poor resolutions are prone to be inaccurate, especially within the most flexible regions, such as loops^29,31^ or gating residues within tunnels.^3,5^ Therefore, in some cases, a simple analysis of crystal structure leads to an incomplete picture of the enzyme’s tunnel network.

CAVER software ranks the tunnels identified in crystal structures according to their priority, which is computed by averaging tunnel throughput, which is a measure of the cost of each pathway and can range from 0 (worst) to 1 (best). For tunnels identified during MD simulation, priority was averaged over all MD simulation frames in which a tunnel was identified. Analysis of the tunnel ranking showed no correlation between crystal structures and MD simulations. The differences in tunnel ranking between crystal structures and MD simulations may be associated with several factors, e.g., dense packing of the structures in crystals or multiple conformations that are accessible during MD simulation. However, comparison of the maximal bottleneck radii showed good correlation between the corresponding tunnels in crystal structures and MD simulations (*r* = 0.82) (Figure S1). On average, the difference between the measured bottleneck radii of the crystal structure was smaller than the bottleneck measured during the MD simulation by about 0.7 Å.

A high correlation between measured bottlenecks in corresponding tunnels from the crystal structure and MD simulations may suggest that the shape and size of the tunnels present in a crystal structure are preserved despite the potential conformational changes which may affect overall protein structure. However, closer analysis of the tunnels identified in crystal structures and during MD simulations by CAVER showed that the tunnels identified in crystal structures are well-defined; however, their parts located closer to the protein surface are, in some cases, coiled. For most tunnels identified during MD simulations, the interior parts of tunnels were well-defined, whereas the tunnels’ mouths were widely distributed on the protein surface. Such an observation might suggest that those regions are tightly packed and/or lined by bulky residues, which can change their conformation to open/close a particular tunnel. Therefore, we recommend tunnel identification using MD simulations instead of a single crystal structure. However, the geometry-based approach has issues related to the asymmetrical shape of the tunnel: multiple tunnels identified by CAVER during MD simulations may in fact be the same tunnel, as it was shown in the case of the Tc/m tunnel (Figure S2). Part of the tunnels can be seen as short-lasting cavities, which rarely connect with other internal voids,^17^ and as such, they are difficult to identify using the geometry-based approach.

### Comparison of the Results Obtained with Geometry-Based and Small-Molecule Tracking Approaches from MD Simulations

Using the same MD simulations as an input for two different approaches provided an opportunity to compare the results. While CAVER was developed to find all possible entrances to the enzymes’ active sites, defined as a space accessible for the probe with a defined size in particular frames, AQUA-DUCT is focused on the tunnel’s functionality, defined as the ability of a tunnel (or cavity) to transport small molecules of interest. However, we observed that both tools were able to identify the main tunnels (the most often open/the most used by water molecules) in the analyzed sEHs. The difference between the approaches is more visible when comparing the side tunnels (rarely open/used by less water molecules). Here, we would like to point out that the aim of the study was not only to compare both approaches but also to equip the user with a set of guidelines on how to carefully interpret the information on the tunnel network provided by each tool. We noticed that in several cases AQUA-DUCT was unable to detect tunnels identified by the geometry-based approach in both crystal structure and during MD simulation. This may be caused by the physicochemical properties of the tunnel-lining residues, which could block the transport of particular molecules via the selected pathway. According to our analysis, such nonfunctional tunnels were rather common, not rare, cases. They were found by CAVER 3.0 PyMOL plugin and CAVER 3.02 in seven out of eight analyzed sEHs (all except msEH). Such tunnels may not be used for the transport of small molecules; however, their modification can lead to improved (thermo)stability of the protein.^32^ We also noticed tunnels which were identified by AQUA-DUCT but not by the geometry-based approach. At first glance, such a finding for MD simulation analysis is unexpected because of the effective radius of the water molecule, which was bigger than the probe used in our investigation. However, this can be observed due to two factors: (i) “rare events” or “water leakage” and (ii) clustering algorithm. Rare events were previously discussed in the case of StEH1.^33^ Rare events can be identified by AQUA-DUCT even during relatively short MD simulations (50 ns), but their identification by CAVER may be challenging. When a water molecule is transported from one internal cavity to another during the course of an MD simulation, it can leak through the protein region, which is equipped with a set of connected cavities and not a permanent tunnel (which is a must for a geometry-based approach). Protein motions can promote molecule passage, and therefore, longer or advanced sampling MD simulations need to be performed to detect such a void continuum by a geometry-based approach. Another option is to use a smaller probe, which will make computations longer and analysis more challenging. The clustering approach used by CAVER searched for the similarities between detected pathways, and thus, it omitted the rarely occurring tunnels. Therefore, we recommend a careful analysis of the clustering results. It is worth noting that tunnels which were identified by AQUA-DUCT and not by CAVER 3.02 during MD simulations may be used for modifying an identified “rare event” tunnel whose opening may lead to improved protein activity^34−36^ or selectivity.^37−40^ Importantly, AQUA-DUCT is designed to track all types of small molecules,^11,24^ such as water molecules, ions, ligands, and additional cosolvents, such as methanol, urea, dimethyl sulfoxide, acetonitrile, or phenol (the use of AQUA-DUCT for the analysis of cosolvents was shown by Bzówka et al.^41^). For this study, we used water molecules; because of the analyzed system, sEHs require a catalytic water molecule to convert substrates to product(s), and therefore, they should be equipped in tunnels maintaining water molecule transport. However, in the case of proteins whose tunnels transport hydrophobic molecules, such as AlkL, which was proposed to facilitate a passive transport function increasing the rate of alkane diffusion,^42^ using a hydrophobic probe would be recommended.

**Figure 3:**
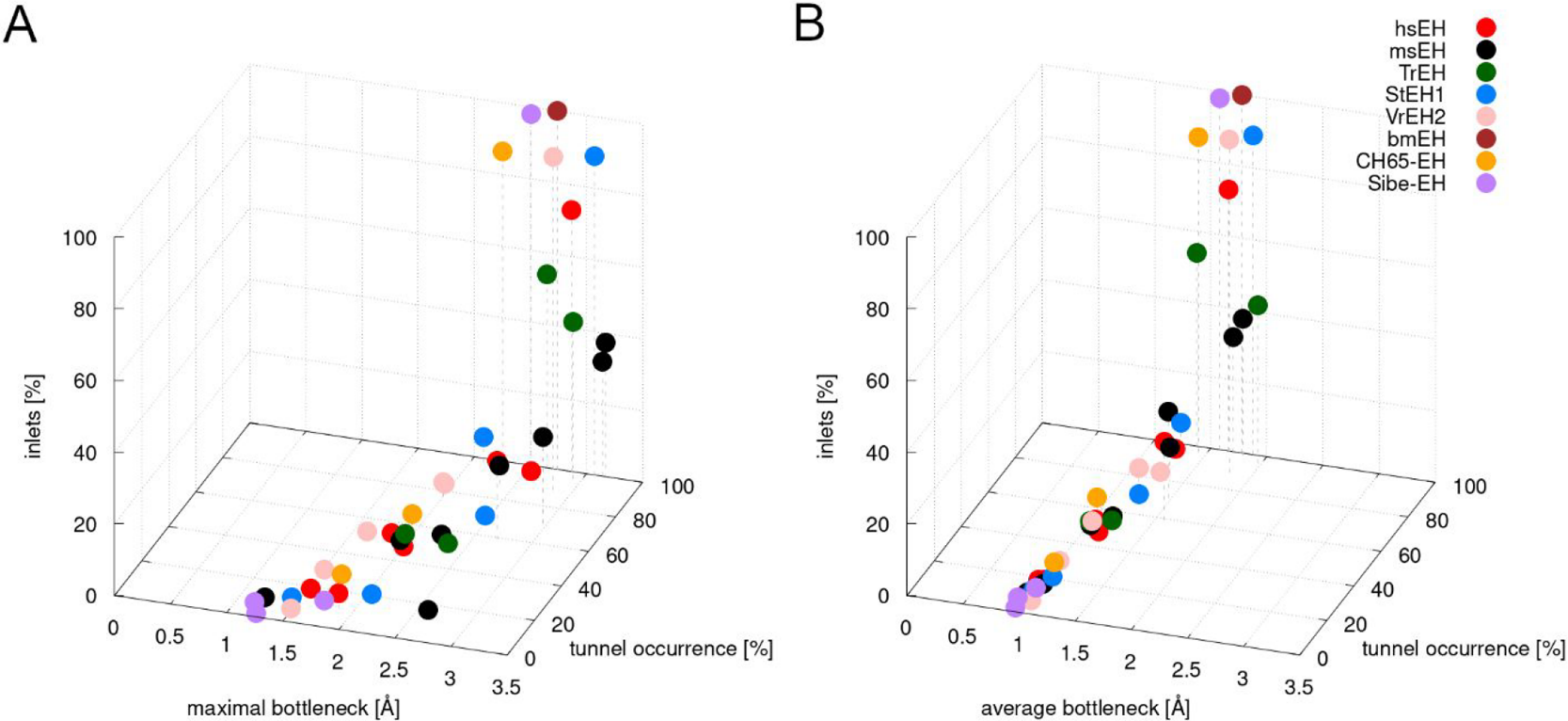
Relationship between number of inlets, tunnel occurrence, and average and maximal bottleneck obtained for all analyzed proteins. Please note that even those tunnels which were always open and had wide bottlenecks were not always identified as functional tunnels.

A comparison of the geometry-based and the small-molecule tracking approaches also showed other differences. Tunnels identified by the small-molecule tracking approach can be considered functional for particular types of traced molecules. The number of molecules passing through a particular entry/exit reflects the tunnels’ usability, whereas in the case of tunnels identified by the geometry-based approach, we were unable to determine the tunnels’ functionality. Taking into account all analyzed sEHs, no correlation between the number of inlets and average bottleneck radius, maximal bottleneck radius, or tunnel occurrence was found (Figure 3). However, for particular enzymes, such correlation can be observed. In msEH, both Tc/m and Tm1 tunnels have similar bottleneck radii and occurrence according to the CAVER 3.02 results (Table 1), and AQUA-DUCT showed that they are used to transport 38% and 32% of the identified water molecules, respectively. Interestingly, the Tg tunnel, which was not present in the crystal structure, also has a similar bottleneck radius but occurred only in 71% of the simulation time. The Tg tunnel was used to transport around 24% of the identified water molecules. All above-mentioned tunnels can be considered similar. In contrast, in StEH1, the Tm1 and Tm2 tunnels which have similar maximal bottlenecks (>2.5 Å) differ substantially in terms of functionality: Tm1 was used by 89% of water molecules and Tm2, by 3%. On the other hand, the Tm1 and Tc/m1 tunnels were almost always open, and they also differed in terms of their functionality (Tc/m1 was used by 7% of water molecules).

In this study, we compared the software for tunnel identification, namely, the geometry-based approach of CAVER 3.02 and small-molecule tracking approach of AQUA-DUCT. We used the crystal structures and a set of MD simulation trajectories to compare the results. Moreover, we wanted to raise awareness among the users of tunnel identification software that the geometry-based approach has its flaws, which may be overcome or supplemented by using the small-molecule tracking approach. Even though the main tunnels in the analyzed proteins seem to be described in a comparable way by both approaches, the results differ when it comes to the side tunnels, which can be of great importance for catalysis. Those differences are related to the way in which both approaches search for tunnels. However, it must be kept in mind that we were analyzing tunnels with relatively narrow bottlenecks (1.0–2.0 Å radii) in which a subtle conformational change may cause opening or closing of a particular pathway. Therefore, the described differences between the approaches may not be observable in wider tunnels and channels. We also showed that MD simulations provide much more information on the tunnels and protein dynamics. The small-molecule tracking approach was shown to solve some limitations of the geometry-based approach; however, in some aspects, both approaches are complementary and may be useful for further protein engineering. Because tunnel detection in the crystal structures using the geometry-based approach is easier compared with other approaches using MD simulation data, it may be the most commonly used. We hope that our work will increase awareness among researchers using a geometry-based approach about its limitations and will provide a guide for the selection of methods according to their needs.

## Data and Software Availability

Tunnel identification in the crystal structures was carried out using a CAVER 3.0 plugin for PyMOL.^23^ Parameters used for the analysis are specified in Table S1. The classical MD simulations of each protein were carried out using the CUDA version of the pmemd program available in Amber14.^21^ Tunnel identification during MD simulations was performed by CAVER 3.02 software^23^ using the same set of parameters as for the analysis in the crystal structures (as in Table S1) and using AQUA-DUCT 1.0 version.^11,24^ For AQUA-DUCT analysis, the water molecules which entered and/or left the active site cavity (called the Object) were traced within the protein’s interior (called the Scope).

## Supporting Information Available

Number of added ions and water molecules along with the protonation pH and the duration of the equilibration step for each of the analyzed systems; list of parameters set; comparison of tunnels identified with the geometry-based approach in both crystal structure and during MD simulation for all analyzed proteins; correlation between maximal bottleneck radii measured in corresponding tunnels identified in both the crystal structure and in the MD simulation for each protein structure; comparison between Tc/m tunnels identified by CAVER 3.02 software during MD simulations and the cluster of inlets identified by AQUA-DUCT (PDF)

## Abbreviations

sEH: soluble epoxide hydrolase
hsEH: human soluble epoxide hydrolase
msEH: mouse soluble epoxide hydrolase
TrEH: *Trichoderma reesei* soluble epoxide hydrolase
StEH1: *Solanum tuberosum* soluble epoxide hydrolase
VrEH2: *Vigna radiata* soluble epoxide hydrolase
bmEH: *Bacillus megaterium* soluble epoxide hydrolase
Sibe-EH: soluble epoxide hydrolase from hot springs in Russia
CH65-EH: soluble epoxide hydrolase from hot springs in China
MD: molecular dynamics
PDB: Protein Data Bank
