# Supplementary material for: Geometry-Based versus Small-Molecule Tracking Method for Tunnel Identification: Benefits and Pitfalls
Source: J Chem Inf Model. 2022 Nov 14;62(24):6803–11. doi: 10.1021/acs.jcim.2c00985 (PMC9795556; doi:10.1021/acs.jcim.2c00985)

Geometry-based versus small-molecule tracking method for tunnel identification – benefits and pitfalls

*Karolina Mitusińska‡, Maria Bzówka‡, Tomasz Magdziarz, Artur Góra**

Tunneling Group, Biotechnology Centre, Silesian University of Technology, Krzywoustego 8, 44-100 Gliwice, Poland

Corresponding Author

* E-mail: a.gora@tunnelinggroup.pl

Author Contributions

‡These authors contributed equally.

Supplementary Information

Supplementary Table S1. The number of added ions, water molecules, along with the protonation pH and the duration of the equilibration step for each of the analysed systems.

Supplementary Table S2. The list of parameters set for both CAVER plugin and CAVER 3.0 tunnels identification for each of the analyzed systems.

Supplementary Table S3. Comparison of tunnels identified with the geometry-based approach in both crystal structure and during MD simulation for hsEH.

Supplementary Table S4. Comparison of tunnels identified with the geometry-based approach in both crystal structure and during MD simulation for msEH.

Supplementary Table S5. Comparison of tunnels identified with the geometry-based approach in both crystal structure and during MD simulation for TrEH.

Supplementary Table S6. Comparison of tunnels identified with the geometry-based approach in both crystal structure and during MD simulation for StEH1.

Supplementary Table S7. Comparison of tunnels identified with the geometry-based approach in both crystal structure and during MD simulation for VrEH2.

Supplementary Table S8. Comparison of tunnels identified with the geometry-based approach in both crystal structure and during MD simulation for bmEH.

Supplementary Table S9. Comparison of tunnels identified with the geometry-based approach in both crystal structure and during MD simulation for Sibe-EH.

Supplementary Table S10. Comparison of tunnels identified with the geometry-based approach in both crystal structure and during MD simulation for CH65-EH.

Supplementary Figure S1. Correlation between maximal bottleneck radii measured in corresponding tunnels identified in both the crystal structure and in the MD simulation for each protein structure.

Supplementary Figure S2. Comparison between Tc/m tunnels identified by CAVER 3.02 software during MD simulations and the cluster of inlets identified by AQUA-DUCT.

Supplementary Table S1. The number of added ions, water molecules, along with the protonation pH and the duration of the equilibration step for each of the analysed systems.

PDB ID	Num. of added ions	Num. of added water molecules	protonation pH	equilibration duration
**1cqz**	1 Cl-	8659	7.0	1000ps
**1s8o**	-	9883	6.5	5000ps
**2cjp**	5 Na+	9362	6.8	1000ps
**5uro**	4 Na+	9980	6.5	1000ps
**4nzz**	9 Na+	8548	6.5	1000ps
**5ng7**	3 Cl-	10738	6.5	5000ps
**5nfq**	4 Na+	10569	6.5	5000ps
**5xm6**	-	8706	6.5	5000ps

Supplementary Table S2. The list of parameters set for both CAVER 3.0 PyMOL plugin and CAVER 3.02 tunnels identification for each of the analyzed systems.

	shell_radius	shell_depth	probe_radius	clustering_threshold
hsEH	4	6	0.9	4
msEH	4	6	0.9	5
TrEH	3	4	0.9	4
StEH1	4	5	0.9	4
VrEH2	4	3	0.9	3.5
bmEH	4	6	0.9	5
Sibe-EH	4	5	0.9	4
CH65-EH	5	5	0.9	4

Supplementary Table S3: Comparison of tunnels identified with the geometry-based approach in both crystal structure and during MD simulation for hsEH. mdX stands for tunnels identified during MD simulations, pluginX – for tunnels identified in crystal structures, and X stands for the ID of the identified tunnel.

	plugin1	plugin2	plugin3	plugin4	plugin5	plugin6	plugin7	plugin8	plugin9
**md1**	0.6364	**0.1250**	0.5714	0.6522	0.4138	0.3333	0.6905	0.5610	0.6667
**md2**	**0.1000**	0.5882	0.3889	0.4286	0.6957	0.5484	0.6216	0.7111	0.7714
**md3**	0.7321	0.3214	0.6981	0.7414	0.3939	0.5000	0.7647	0.6316	0.7045
**md4**	0.2703	0.6818	0.5250	0.5652	0.7313	0.6410	0.6889	0.6757	0.7604
**md5**	0.7273	0.4000	0.6923	0.7368	**0.2881**	0.5686	0.7636	0.6727	0.7353
**md6**	0.5854	0.2188	0.5000	0.6047	0.5111	0.3846	0.6829	0.5000	0.6216
**md7**	0.5000	0.6429	0.4194	0.4412	0.7258	0.6071	0.4054	0.7377	0.7838
**md8**	0.4000	0.4706	**0.1724**	0.4167	0.6389	0.4583	0.5758	0.6538	0.7231
**md9**	0.5745	0.6818	0.5385	0.4318	0.7407	0.6410	**0.2059**	0.7547	0.8030
**md10**	0.6250	0.2424	0.5676	0.6429	0.4828	0.4000	0.6750	0.5250	0.6415
**md11**	0.7091	0.3250	0.6731	0.7193	0.3968	0.5745	0.7547	0.6471	0.7188
**md12**	0.1000	0.6316	0.4118	0.4318	0.7083	0.5758	0.6410	0.7234	0.7833
**md13**	0.4839	0.6596	0.4643	0.3333	0.7193	0.6190	0.4444	0.7321	0.7826
**md14**	0.6170	0.4634	0.6304	0.6863	0.6226	0.5385	0.7400	**0.3333**	0.5000
**md15**	0.2000	0.6250	0.3514	**0.2683**	0.7209	0.5938	0.6216	0.7391	0.7901
**md16**	0.6667	0.3438	0.6222	0.6800	0.4219	**0.2353**	0.7222	0.6200	0.6984
**md17**	0.6667	0.3438	0.6000	0.6818	0.4912	0.4667	0.7143	0.5814	0.6786
**md18**	0.6923	0.4000	0.6522	0.7037	0.3191	0.3864	0.7347	0.6400	0.7143
**md19**	0.5769	0.6852	0.5200	0.5385	0.7344	0.5862	0.4091	0.7460	0.7875
**md20**	0.5294	0.2857	0.4091	0.5556	0.5417	0.3704	0.6286	0.5366	0.6481

Supplementary Table S4: Comparison of tunnels identified with the geometry-based approach in both crystal structure and during MD simulation for msEH. mdX stands for tunnels identified during MD simulations, pluginX – for tunnels identified in crystal structures, and X stands for the ID of the identified tunnel.

	**plugin1**	**plugin2**	**plugin3**	**plugin4**	**plugin5**	**plugin6**
**md1**	**0.1111**	0.5938	0.6984	0.7361	0.6316	0.4545
**md2**	0.6500	**0.2500**	0.3824	0.4324	0.5000	0.7627
**md3**	0.5909	0.5714	0.6667	0.7101	**0.2500**	0.6716
**md4**	0.3448	0.4865	0.6122	0.6724	0.4634	0.6389
**md5**	0.7258	0.4583	0.4143	**0.1667**	0.6406	0.7901
**md6**	0.6750	0.2857	**0.3636**	0.4531	0.5510	0.7703
**md7**	0.3158	0.6250	0.7143	0.7500	0.6667	0.6571
**md8**	0.4286	0.6364	0.7273	0.7600	0.6842	0.6667
**md9**	0.6078	0.6727	0.7246	0.7564	0.6545	**0.3636**
**md10**	0.6458	0.5000	0.6000	0.6104	0.2826	0.7463
**md11**	0.8000	0.5397	0.4658	0.5696	0.7222	0.8403
**md12**	0.6667	0.6857	0.7324	0.7708	0.7317	0.7656
**md13**	0.6552	0.7143	0.7794	0.8000	0.6979	0.6883
**md14**	0.7627	0.4872	0.4565	0.3571	0.6533	0.8193

Supplementary Table S5: Comparison of tunnels identified with the geometry-based approach in both crystal structure and during MD simulation for TrEH. mdX stands for tunnels identified during MD simulations, pluginX – for tunnels identified in crystal structures, and X stands for the ID of the identified tunnel.

	plugin1	plugin2	plugin3	plugin4	plugin5	plugin6	plugin7	plugin8
**md1**	**0.1429**	0.4348	0.5333	0.4815	0.6364	0.6190	0.6667	0.6923
**md2**	0.3333	**0.1579**	0.3478	0.5862	0.4194	0.7000	0.4853	0.7500
**md3**	0.5556	0.3333	**0.1463**	0.6842	0.4219	0.7551	0.4789	0.7895
**md4**	0.5909	0.6226	0.6667	**0.2174**	0.7260	0.5072	0.7500	0.5570
**md5**	0.3125	0.2500	0.3750	0.5625	0.5161	0.6429	0.5588	0.7000
**md6**	0.5000	0.1500	0.4231	0.6667	**0.3514**	0.7333	0.5429	0.7714
**md7**	0.6000	0.3478	0.5122	0.7073	0.5882	0.6575	0.6267	0.7614
**md8**	0.4762	0.5600	0.6053	0.6563	0.6944	0.6923	0.7143	0.7419
**md9**	0.2778	0.2632	0.3913	0.5517	0.5313	0.6410	0.5714	0.7021
**md10**	0.5517	0.4545	0.5778	0.6750	0.6545	0.6119	0.6825	0.7143
**md11**	0.4483	0.6410	0.6818	0.4250	0.7358	0.6389	0.7600	0.6707
**md12**	0.5500	0.6000	0.6552	0.6957	0.7222	0.7143	0.7436	0.7576
**md13**	0.6757	0.5926	0.6125	0.7500	0.6067	0.7797	**0.4632**	0.8060
**md14**	0.2857	0.3000	0.2051	0.5833	0.5238	0.6522	0.5075	0.7143
**md15**	0.4615	0.6842	0.7209	0.6316	0.7667	0.7500	0.7879	0.7838

Supplementary Table S6: Comparison of tunnels identified with the geometry-based approach in both crystal structure and during MD simulation for StEH1. mdX stands for tunnels identified during MD simulations, pluginX – for tunnels identified in crystal structures, and X stands for the ID of the identified tunnel.

	plugin1	plugin2	plugin3	plugin4	plugin5	plugin6	plugin7
**md1**	**0.1842**	0.4474	0.3125	0.7143	0.7097	0.7407	0.7143
**md2**	0.2381	0.5455	**0.1471**	0.7407	0.7534	0.7627	0.7407
**md3**	0.4091	**0.1034**	0.5208	0.7000	0.6607	0.7273	0.7000
**md4**	0.4231	0.6226	0.3529	0.7778	0.7692	0.7941	0.7778
**md5**	0.3250	0.3235	0.4286	0.7021	0.6949	0.7308	0.7021
**md6**	0.4643	0.6491	0.3750	0.7910	0.7931	0.8056	0.7910
**md7**	0.6610	0.5806	0.7077	0.6667	**0.0938**	0.7105	0.4722
**md8**	0.6140	0.5714	0.6724	0.5429	0.5306	0.5946	0.1333
**md9**	0.6429	0.5714	0.6949	0.5152	0.5370	0.5714	**0.1212**
**md10**	0.6129	0.5417	0.6923	0.4231	0.6757	0.5313	0.4839
**md11**	0.6000	0.4524	0.6607	0.6250	0.4565	0.6744	0.5556
**md12**	0.6250	0.5769	0.7000	**0.1154**	0.6923	0.4167	0.5588
**md13**	0.2121	0.5000	0.3810	0.7358	0.7458	0.7586	0.7358
**md14**	0.6216	0.5517	0.6949	0.6471	0.2667	0.6944	0.5000
**md15**	0.6000	0.5000	0.6842	0.4400	0.6452	0.5172	0.3571
**md16**	0.6912	0.6563	0.7324	0.3750	0.7333	**0.1071**	0.6250
**md17**	0.4643	0.6491	0.4182	0.7910	0.7857	0.8056	0.7910
**md18**	0.6129	0.4878	0.6727	0.4615	0.5893	0.5313	0.3548
**md19**	0.5500	0.3333	0.6275	0.5833	0.5818	0.6341	0.5366
**md20**	0.6452	0.6111	0.6984	0.6923	0.3913	0.7308	0.6136

Supplementary Table S7: Comparison of tunnels identified with the geometry-based approach in both crystal structure and during MD simulation for VrEH2. mdX stands for tunnels identified during MD simulations, pluginX – for tunnels identified in crystal structures, and X stands for the ID of the identified tunnel.

	plugin1	plugin2	plugin3	plugin4
**md1**	**0.0500**	0.7692	0.7719	0.4386
**md2**	0.4032	0.8333	0.8293	**0.1667**
**md3**	0.3729	0.8286	0.8250	0.2167
**md4**	0.5714	**0.2083**	0.5476	0.6364
**md5**	0.5588	0.5556	0.2500	0.6364
**md6**	0.2353	0.7937	0.8088	0.3929
**md7**	0.6129	0.2857	0.5870	0.6667
**md8**	0.4590	0.8060	0.8182	0.1964
**md9**	0.5789	0.3438	0.4878	0.6418
**md10**	0.5769	0.4688	0.6500	0.6452
**md11**	0.3704	0.8060	0.8028	0.3898
**md12**	0.1000	0.7833	0.7797	0.3443
**md13**	0.4219	0.8243	0.8354	0.3284
**md14**	0.3968	0.8333	0.8293	0.2222
**md15**	0.6000	0.6923	0.6364	0.6571
**md16**	0.3729	0.8116	0.8243	0.1754
**md17**	0.3393	0.8030	0.8000	0.2182
**md18**	0.5556	0.4412	0.4800	0.6250
**md19**	0.6349	0.5909	**0.2414**	0.6849
**md20**	0.4262	0.8116	0.8082	0.2807

Supplementary Table S8: Comparison of tunnels identified with the geometry-based approach in both crystal structure and during MD simulation for bmEH. mdX stands for tunnels identified during MD simulations, pluginX – for tunnels identified in crystal structures, and X stands for the ID of the identified tunnel.

	**plugin1**	**plugin2**	**plugin3**	**plugin4**
**md1**	0.1667	**0.0625**	0.4706	0.5897
**md2**	0.6667	0.6429	0.3721	0.7759
**md3**	0.6400	0.6250	**0.1111**	0.7805
**md4**	0.6552	0.6563	0.7500	**0.5152**

Supplementary Table S9: Comparison of tunnels identified with the geometry-based approach in both crystal structure and during MD simulation for Sibe-EH. mdX stands for tunnels identified during MD simulations, pluginX – for tunnels identified in crystal structures, and X stands for the ID of the identified tunnel.

	**plugin1**	**plugin2**	**plugin3**	**plugin4**	**plugin5**	**plugin6**
**md1**	**0.0526**	0.3462	0.4783	0.3864	0.4839	0.6190
**md2**	0.6585	0.6739	0.3548	0.4500	0.7347	0.7833
**md3**	0.3478	**0.0800**	0.5400	0.4583	0.5484	0.6364
**md4**	0.3462	0.5000	0.5102	0.3913	0.5161	0.6429
**md5**	0.5714	0.5641	0.6765	0.6418	0.6744	0.5217
**md6**	0.5789	0.6667	0.3400	**0.0571**	0.7292	0.7797
**md7**	0.4667	0.5946	0.4000	0.3056	0.6591	0.7037
**md8**	0.6809	0.6923	0.7551	0.7284	0.7455	0.6774
**md9**	0.6939	0.7273	**0.2000**	0.4098	0.7759	0.8116
**md10**	0.7321	0.7377	0.4559	0.5224	0.7813	0.8133
**md11**	0.7288	0.7500	0.2667	0.5156	0.7937	0.8193
**md12**	0.3000	0.4583	0.5714	0.4717	**0.4231**	0.5385
**md13**	0.5385	0.5000	0.7105	0.6622	0.4524	**0.2326**
**md14**	0.6889	0.7000	0.3167	0.3111	0.7547	0.7969
**md15**	0.7500	0.7746	0.4507	0.5417	0.8108	0.8295

Supplementary Table S10: Comparison of tunnels identified with the geometry-based approach in both crystal structure and during MD simulation for CH65-EH. mdX stands for tunnels identified during MD simulations, pluginX – for tunnels identified in crystal structures, and X stands for the ID of the identified tunnel.

	plugin1	plugin2	plugin3
**md1**	0.6604	**0.1591**	0.7368
**md2**	0.1765	0.5000	0.4375
**md3**	0.3043	0.5135	0.3125
**md4**	0.7069	0.2703	0.7727
**md5**	0.4615	0.5909	**0.1739**
**md6**	0.6600	0.4808	0.7358
**md7**	**0.1579**	0.5610	0.5000
**md8**	0.5517	0.6000	0.4375
**md9**	0.6792	0.6842	0.6667
**md10**	0.6667	0.3273	0.7414
**md11**	0.6383	0.6604	0.6000
**md12**	0.7119	0.7195	0.7083
**md13**	0.7344	0.3509	0.7941
**md14**	0.6596	0.5600	0.7368
**md15**	0.7377	0.7368	0.7308

Supplementary Figure S1. Correlation between maximal bottleneck radii measured in corresponding tunnels identified in both the crystal structure and during MD simulation for each protein structure.

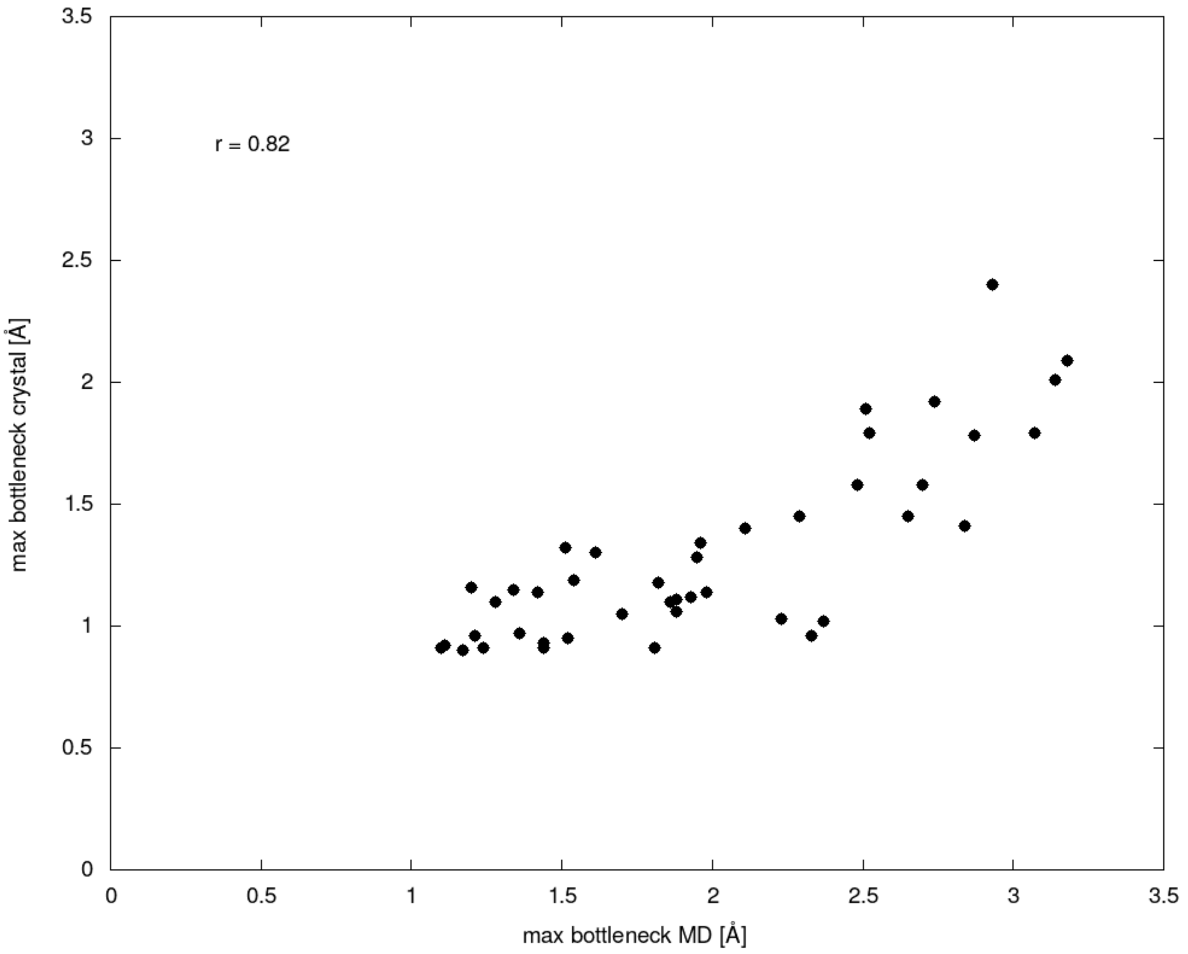

Supplementary Figure S2. Comparison between Tc/m tunnels identified by CAVER 3.02 software during MD simulations (shown as lines) and the cluster of inlets identified by AQUA-DUCT (shown as green spheres). The Tc/m1 tunnel is shown as dark green centerlines, the Tc/m2 tunnel - green, and the Tc/m3 tunnel - light green. The protein is shown in cartoon representation. Please note that the inlets cover a large area of the protein surface while tunnels are narrower. Also, please note the branch located in the middle of the dark green tunnel.

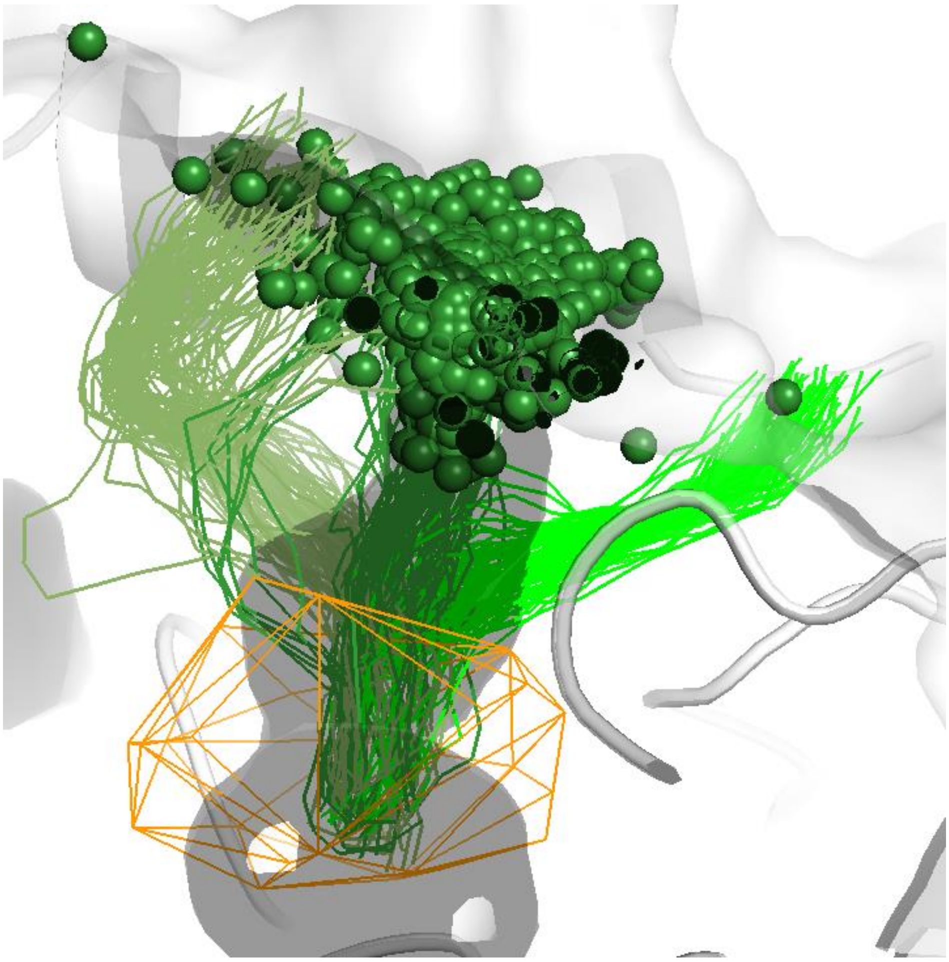

Supplement: Supplementary file 1 — ci2c00985_si_001.pdf [file ci2c00985_si_001.pdf]
